# Sarcopenic obesity and risk of new onset depressive symptoms in older adults: English Longitudinal Study of Ageing

**DOI:** 10.1038/ijo.2015.124

**Published:** 2015-09-08

**Authors:** M Hamer, G D Batty, M Kivimaki

**Affiliations:** 1 Department of Epidemiology and Public Health, University College London, London, UK

## Abstract

**Background:**

We examined the role of sarcopenic obesity as a risk factor for new-onset depressive symptoms over 6-year follow-up in a large sample of older adults.

**Methods:**

The sample comprised 3862 community dwelling participants (1779 men, 2083 women; mean age 64.6±8.3 years) without depressive symptoms at baseline, recruited from the English Longitudinal Study of Ageing. At baseline and 4-year follow-up, handgrip strength (kg) of the dominant hand was assessed using a hand-held dynamometer, as a measure of sarcopenia. The outcome was new onset depressive symptoms at 6-year follow-up, defined as a score of ⩾4 on the 8-item Centre of Epidemiological Studies Depression scale. Sarcopenic obesity was defined as obese individuals (body mass index ⩾30 kg m^−^^2^) in the lowest tertile of sex-specific grip strength (<35.3 kg men; <19.6 kg women).

**Results:**

Using a multivariable logistic regression model, the risk of depressive symptoms was greatest in obese adults in the lowest tertile of handgrip strength (odds ratio (OR), 1.79, 95% confidence interval (CI), 1.10, 2.89) compared with non-obese individuals with high handgrip strength. Participants who were obese at baseline and had a decrease of more than 1 s.d. in grip strength over 4-year follow-up were at greatest risk of depressive symptoms (OR=1.97, 95% CI, 1.22, 3.17) compared with non-obese with stable grip strength.

**Conclusions:**

A reduction in grip strength was associated with higher risk of depressive symptoms in obese participants only, suggesting that sarcopenic obesity is a risk factor for depressive symptoms.

## Introduction

The association between two disorders of major public health importance, obesity and mental health, remains unclear.^1, 2, 3, 4, 5, 6^ A recent meta-analysis containing prospective cohort studies showed an association between body mass index (BMI) and risk of depressive symptoms,^1^ although some individual studies have found no association^2^ and others suggest that greater body weight may actually confer protection against future mental health problems and suicide.^3, 4^ Furthermore, the discordant results remain unclear when using an unconfounded instrument variable for obesity (adiposity-related genetic variants).^5, 6^

Different obesity phenotypes may exist,^7^ and in particular variation in skeletal muscle mass across obese individuals may confer different health risks.^8^ Sarcopenia, a syndrome characterized by a progressive loss of skeletal muscle mass and quality (or strength) resulting in impaired physical performance, is related to an increased risk of physical disability, poor quality of life and death.^9^ Sarcopenic obesity, a co-occurrence of low muscle strength and excess body fat, is an emerging clinical entity in which these two states are thought to act in negative synergism in the pathophysiology of both metabolic, functional impairments and mortality risk.^8, 10, 11, 12, 13, 14, 15, 16, 17^ Although sarcopenia appears to be associated with cognitive impairment and depressive symptoms in the few studies conducted,^18, 19^ these were small in scale (resulting in modest statistical power), based on non-representative samples (reducing generalisablity) and, most importantly, cross-sectional (hampering insights into the directionality of any relationships).

The aim of this study was to examine the role of sarcopenic obesity as a risk factor for new-onset depressive symptoms over 6-year follow-up in a large, well characterized, general population-based sample of older adults. Since the skeletal muscle is known to have a role in various metabolic responses, we hypothesised sarcopenia could adversely affect mental function via metabolic and endocrine mechanisms. Our hypothesis was that obesity and sarcopenia in combination are associated with a greater depression risk than either of the disorders alone.

## Materials and methods

### Study sample and procedures

The English Longitudinal Study of Ageing is an ongoing cohort study of a nationally representative sample of the English population born on or before 29 February 1952 living in private households.^20^ A multistage stratified probability sampling method using postcode sectors and household addresses was used to recruit the sample. Participants gave full, informed written consent to take part in the study and ethical approval was obtained from the London Multicentre Research Ethics Committee. For the purposes of the present analyses, data collected in 2004/05 (wave 2) were used as the baseline, as this was the first occasion on which clinical information was gathered, and follow-up for new cases of depression was performed in 2010/11. The inclusion criteria were absence of depression at baseline and availability of exposure, outcome and covariate data. For the key exposure measure, grip strength, there were no upper age limits although respondents were excluded if they had swelling or inflammation, severe pain or a recent injury or surgery to the hand in the preceding 6 months. Data on the key exposure variables at baseline were available in 7055 participants, although 557 were excluded at baseline with depressive symptoms, a further 2028 were lost to follow-up (due to death, emigration, institutionalisation), and 608 participants had missing data on covariables or outcome. Thus, the final analytic sample consisted of 3862 individuals.

### Measures

#### Exposure

Handgrip strength (kg) of the dominant hand was assessed using the Smedley hand-held dynamometer (Stoelting Co, IL, USA), using the average of three measurements. Participants were required to hold the device at a right angle to their body and exert maximum force for a couple of seconds when instructed. Successive trials were alternated between dominant and non-dominant hands. Nurses measured participants' body weight to the nearest 0.1 kg using Tanita electronic scales (Tanita Co, IL, USA) without shoes and in light clothing, and height was measured using a Stadiometer with the Frankfurt plane in the horizontal position; BMI was calculated using the standard formula (weight (kg)/height^2^ (m^2^)). Clinical data were collected in wave 2 (2004/5) and wave 4 (2008/9) that also allowed us to examine changes in grip strength and body mass.

#### Covariables

At baseline, trained interviewers collected information on psychosocial, demographic and health-related factors. These questions included self-reported cigarette smoking (current, previous or non-smoker), the self-reported frequency of participation in vigorous, moderate and light physical activities (more than once per week, once per week, one to three times per month, hardly ever), self-reported frequency of alcohol intake (daily, 5 to 6/week, 3 to 4/week, 1 to 2/week, 1 to 2/month, once every couple of months, 1 to 2/year, never) and self-reported physician-diagnosed medical conditions (cardiovascular diseases, diabetes, cancer, arthritis). Participants reported new onset of these diseases every 2 years through follow-up thus we were able to derive a time-varying accumulative chronic disease variable. Self-reported wealth was used as our measure of socioeconomic status. The wealth variable comprised the total value of the participant's home (excluding mortgage), financial assets such as savings, business assets and physical wealth such as artwork or jewellery, which has been shown to best capture the material resources available to older adults.^21^

#### Outcome

Depressive symptoms were assessed at baseline and follow-up using the self-reported 8-item Centre of Epidemiological Studies Depression scale. As in previous studies, we used a score of ⩾4 to define cases of elevated depressive symptoms.^22^ Participants scoring ⩾4 at baseline were excluded from the analyses. The Centre of Epidemiological Studies Depression is highly validated for use in older adults, displaying excellent psychometric properties.^23, 24^

### Statistical analysis

Several tests were used (*χ*2 and analysis of variance with Scheffe post-hoc tests) to examine differences in baseline characteristics with respect to new onset depressive symptoms. We used multiple logistic regression to calculate odds ratios (OR) and 95% confidence intervals (CI) for the risk of new cases of elevated depressive symptoms at follow-up in relation to obesity and grip strength (individually and in combination). In preliminary analyses, there was no evidence of effect modification according to gender in the obesity/sarcopenia relation with depressive symptoms; as such, data for men and women were pooled and gender-adjusted. In order to examine additive effects of grip strength and obesity, we categorized the data into sex-specific tertiles of handgrip strength in relation to obesity^1^ (non-obese <30 kg m^−^^2^ and obese⩾30 kg m^−2^). In multivariable models we adjusted for several covariates in a step-wise fashion: Model 1 contained age and sex; and Model 2 contained additional behavioural and clinical covariates, including smoking, alcohol, physical activity, wealth, time-varying accumulative chronic disease through follow-up (cardiovascular disease, diabetes, cancer and arthritis). We chose this modelling strategy *a priori* based on existing data on obesity and depressive symptoms.^1, 22^ We performed sensitivity analyses making further adjustment for metabolic risk factors in a sub-sample of participants with available biomarker data (for a full description of methodology on biomarker data collection see Hamer *et al.*^22^). In addition we studied the association of changes in grip strength with risk of depressive symptoms using the same modelling strategy described above. All analyses were conducted using SPSS (version 20, Chicago, IL, USA).

## Results

The analytic sample comprised 3862 study participants (1779 men, 2083 women; mean age at baseline 64.6±8.3 years). In comparison with the overall baseline sample (excluding participants removed with depression), the sub-group used in the present analyses were younger (68.9 vs 64.6 years, *P*<0.001), had higher mean grip strength (28.3 vs 30.4 kg, *P*<0.001), and better health behaviours including lower rates of smoking (19.1 vs 14.8%, *P*<0.001) and greater physical activity (23.0 vs 32.6%, *P*<0.001, vigorously active ⩾1/week). Although these differences are statistically significant, the absolute difference was small. At 6-year follow-up, there were 328 people with new onset elevated depressive symptoms. In Table 1 we show the characteristics of study participants according to newly developed depression symptoms. Study members with depressive symptoms tended to be older, female, smokers, less physically active, less likely to consume alcohol on a daily basis, be of lower socioeconomic status and have higher prevalence of major chronic somatic diseases.

In models in which ORs were adjusted for multiple covariates (including multiple adjustment for grip strength/obesity depending on the predictor in question), each unit increase in baseline grip strength was associated with lower risk of depressive symptoms (OR=0.98, 95% CI, 0.96–0.99; *P*=0.018) while obesity was related to an elevated risk (OR=1.32, 95% CI, 1.03–1.71; *P*= 0.032).

Compared with non-obese participants with high handgrip strength (our referent group because we anticipated the lowest risk within it), non-obese participants with low handgrip strength experienced an increased risk of depressive symptoms, but the effect estimate was markedly attenuated after multiple adjustments (Table 2). The risk of depressive symptoms in obese adults with low handgrip strength was 1.79 (95% CI, 1.10, 2.89) times greater compared to non-obese individuals with high handgrip strength after multivariate adjustment. Obese participants with high and intermediate handgrip strength demonstrated an increased risk of depressive symptoms, but the effect estimate was markedly attenuated after multiple adjustments. In further analyses we removed participants with BMI<18.5 (*N*=18) although results did not differ.

### Sensitivity analyses

In a slightly smaller sample with data on grip strength at both wave 2 and wave 4 (*n*=3369) it was possible to examine associations of changes in these variables with risk of depressive symptoms. Overall, as anticipated, there was a reduction in grip strength (−1.91±5.7 kg) over the intervening 4 years such that 19.3% of the sample demonstrated more than 1 s.d. reduction. The association between baseline obesity and change in handgrip strength in relation to risk of elevated depressive symptoms at follow-up is presented in Supplementary Table S1. These analyses showed that participants who were obese at baseline and had a decrease of more than 1 s.d. in grip strength were at greatest risk of depressive symptoms (OR=1.97, 95% CI, 1.22, 3.17) compared with non-obese with stable grip strength. In further sensitivity analyses we made adjustment for metabolic risk factors as previously described,^22^ although the pattern of results did not change (Supplementary Table S2). Lastly, we categorised participants using Foundation for the National Institutes of Health (FNIH) sex-specific handgrip cutoffs (men <26 kg; women <16 kg) for those at risk of weakness.^25^ 8.6% of the sample met the threshold for weakness although the pattern of results largely replicated the original results, showing sarcopenic obese were at the highest risk of depressive symptoms (Supplementary Table S3).

## Discussion

This study demonstrates an association between sarcopenic obesity and new onset depressive symptoms in older adults. Hand grip strength, our indicator of sarcopenic loss of muscle mass, was associated with depressive symptoms, an effect that was particularly marked in obese participants. In addition, we were able to show that a reduction in grip strength over 4 years was associated with higher risk of depressive symptoms in obese participants only. Although several small cross-sectional studies have shown associations of sarcopenia with cognitive impairment and depressive symptoms,^18, 19^ this is the first study to examine this issue in obese and non-obese adults longitudinally.

There have been inconsistencies in the pattern of findings across studies relating obesity to mental health outcomes.^1, 2, 3, 4, 5, 6^ We have previously demonstrated that the risk of depressive symptoms is greatest when obesity is combined with an adverse metabolic profile—'metabolically unhealthy obesity'.^22, 26^ The skeletal muscle is known to have a role in various metabolic responses, thus sarcopenia could adversely affect mental function via metabolic and endocrine mechanisms. For example, skeletal muscle represents a major organ for glucose homeostasis, responsible for up to 75% of post-prandial (that is, insulin-stimulated) glucose uptake.^27^ Low muscle mass might therefore be expected to impair glucose homeostasis and various studies have shown a link between glycaemic control and depression.^28, 29, 30^ In previous work on obese adolescents with type 2 diabetes, brain abnormalities, such as reduced white matter volume and enlarged cerebrospinal fluid space, have been found, which might result from changes in vascular function and glucose abnormalities.^31^

As expected, women were more likely to report new onset depressive symptoms than men (covariate adjusted OR= 1.34, 95% CI, 1.04, 1.73). It has been suggested that women may reach a threshold for sarcopenia faster than men, thus the impact of obesity on women may be exaggerated.^17^ This notwithstanding, we found no interaction between muscle strength and sex in relation to new onset depressive symptoms, thus the sex differences may be attributable to other underlying mechanisms.

Presently, there is no consensus on the definition of ‘sarcopenic obesity'. Indeed, the prevalence of sarcopenic obesity depends on the definitions used, and differs considerably when sarcopenia is defined using handgrip strength,^13, 14^ muscle mass^11, 15^ or midarm muscle circumference.^16^ We used handgrip strength as an indicator of sarcopenic loss of muscle mass. Although lean mass and strength (muscle quality) may not decline at the same rate, loss of lean mass is strongly associated with strength decline in both men and women.^32^ The clinical relevance of both measurements is reflected in the revised definition of sarcopenia to incorporate a functional component, that is, to include low muscle strength (muscle quality).^9^ For practical reasons, longitudinal measurements collected in this cohort did not extend to quantification of skeletal muscle mass, but considering the above definition, low handgrip strength might be considered to be equally clinically relevant as formal measurements of low muscle mass. The use of BMI to define obesity in the context of sarcopenia is a further limitation since changes in BMI might partly reflect alterations in lean muscle mass with ageing. English Longitudinal Study of Ageing is designed to be a nationally representative cohort, although the present sample included younger and healthier participants than the overall cohort due to loss of older, more disadvantaged men and women over follow up. Thus the present findings might reflect a conservative estimate of the true effects.

In summary we demonstrate, for the first time, an association between sarcopenia and new onset depressive symptoms that was particularly marked in obese participants. A reduction in grip strength over 4 years was associated with higher risk of depressive symptoms in obese participants only, providing further evidence for sarcopenic obesity and risk of depressive symptoms. Further research is needed to determine whether interventions to maintain muscle quality would reduce depression risk in obese older adults.

## Figures and Tables

**Table 1:** The characteristics of participants according to new onset depressive symptoms at 6-year follow-up

	*Status at follow-up*^a^		
	*Non-depressed (*n=*3534)*	*Depressive symptoms (*n=*328)*	P*-value for difference*
Age at baseline	64.4±8.2	66.5±8.7	<0.001
Sex (%men)	1654 (46.8)	124 (37.8)	<0.001
Cigarette smokers (%)	466 (13.2)	66 (20.1)	<0.001
Vigorous physical activity (%)	1251 (35.4)	78 (23.8)	<0.001
Regular alcohol intake (% daily)	887 (25.1)	78 (23.8)	0.042
Wealth (% in highest quintile)	997 (28.2)	60 (18.3)	<0.001
			
*Prevalent disease*^b^ *(%)*			
Cardiovascular disease/diabetes	1202 (34.0)	158 (48.2)	<0.001
Cancer	399 (11.3)	54 (16.5)	0.002
Arthritis	1467 (41.5)	180 (54.9)	<0.001
Grip strength (kg)	31.2±11.2	27.4±10.5	<0.001
Body mass index (kg m^−2^)	27.8±4.7	28.6±5.4	0.004

a All participants were free of depressive symptoms at baseline.

b Accumulative prevalence from baseline through follow-up. Data presented as mean ±s.d. unless otherwise stated.

**Table 2:** Odds ratios (95% CI) for the association of handgrip strength and obesity at baseline with the risk of new onset elevated depressive symptoms at 6-year follow-up (*n*=3862)

*Handgrip strength*^a^	*Cases/participants at risk*	*Model 1 odds ratio (95% CI)*	*Model 2 odds ratio (95% CI)*
*Non-obese participants (N=2780)*			
High	51/972	1.00 (reference)	1.00 (reference)
Intermediate	76/1010	1.42 (0.98, 2.06)	1.28 (0.87, 1.87)
Low	82/798	1.83 (1.24, 2.70)	1.47 (0.98, 2.20)
			
*Obese participants (N=1082)*			
High	36/423	1.72 (1.11, 2.69)	1.39 (0.88, 2.20)
Intermediate	44/389	2.19 (1.43, 3.35)	1.65 (1.06, 2.58)
Low	39/270	2.60 (1.64, 4.10)	1.79 (1.10, 2.89)

Abbreviation: CI, confidence interval.

Model 1: adjusted for age and sex.

Model 2: adjusted for age, sex, physical activity, smoking, alcohol, wealth, time varying accumulative cardiovascular disease (angina, heart disease, heart failure, heart murmur, arrhythmia and stroke), diabetes, cancer and arthritis.

a In men, the range of handgrip strength at baseline was 4–35.3, 35.4–44.2, >44.2 kg for low, intermediate and high tertiles, respectively. The corresponding ranges in women were 4–19.6, 19.7–24.9, >24.9 kg, respectively.
