# Supplementary material for: Sarcopenic obesity and risk of new onset depressive symptoms in older adults: English Longitudinal Study of Ageing
Source: Int J Obes (Lond). 2015 Sep 8;39(12):1717–20. doi: 10.1038/ijo.2015.124 (PMC4722238; doi:10.1038/ijo.2015.124)
Supplement: Supplementary Information [file ijo2015124x1.docx]

**Online Supplementary material**

**Table S1.** Odds ratios (95% CI) for the association of baseline obesity and change in handgrip strength with risk of new onset elevated depressive symptoms at follow-up (n=3,369)

| **Baseline obesity** | **Grip strength change** | **Cases/People at risk** | **Model 1**  **OR (95% CI)** | **Model 2**  **OR (95% CI)** |
| --- | --- | --- | --- | --- |
| No | Stable | 136/1954 | 1.00 (reference) | 1.00 (reference) |
| No | Decrease | 33/460 | 1.21 (0.81, 1.82) | 1.15 (0.76, 1.74) |
| Yes | Stable | 72/719 | 1.51 (1.12, 2.05) | 1.29 (0.94, 1.77) |
| Yes | Decrease | 27/198 | 2.56 (1.63, 4.04) | 1.97 (1.22, 3.17) |

Model 1: comprises age, sex, baseline grip strength.

Model 2: comprises age, sex, baseline grip strength, physical activity, smoking, alcohol, wealth, time varying accumulative cardiovascular disease (angina, heart disease, heart failure, heart murmur, arrhythmia, stroke), diabetes, cancer, and arthritis.

**Table S2**. Odds ratios (95% CI) for the association of handgrip strength and obesity with risk of new onset elevated depressive symptoms at six years follow-up (n=3,154; a sub-sample with available data on all metabolic risk factors).

| **Handgrip strength** | **Cases/People at risk** | **Model 1**  **OR (95% CI)** | **Model 2**  **OR (95% CI)** |
| --- | --- | --- | --- |
| **Non-obese participants** |  |  |  |
| High | 41/821 | 1.00 (reference) | 1.00 (reference) |
| Intermediate | 64/844 | 1.50 (1.00, 2.27) | 1.33 (0.87, 2.02) |
| Low | 68/645 | 1.96 (1.27, 3.02) | 1.65 (1.05, 2.57) |
| **Obese participants** |  |  |  |
| High | 29/346 | 1.79 (1.09, 2.93) | 1.69 (1.00, 2.84) |
| Intermediate | 36/304 | 2.41 (1.50, 3.87) | 2.13 (1.29, 3.51) |
| Low | 27/194 | 2.62 (1.54, 4.45) | 2.10 (1.19, 3.69) |

Model 1: adjusted for age and sex.

Model 2: adjusted for age, sex, physical activity, smoking, alcohol, wealth, number of metabolic risk factors (hypertension risk [clinic BP >130/85 mmHg, or hypertension diagnosis, or use of anti-hypertensive medication], diabetes risk [HbA1c > 6%], low grade inflammation [CRP≥ 3mg/l], adverse HDL cholesterol profile [<1.03 mmol/l in men and <1.30 mmol/l women], adverse triglycerides [≥ 1.7 mmol/l]), time varying accumulative cardiovascular disease (angina, heart disease, heart failure, heart murmur, arrhythmia, stroke), diabetes, cancer, and arthritis.

**Table S3**. Odds ratios (95% CI) for the association of handgrip strength and obesity with risk of new onset elevated depressive symptoms at six years follow-up using the Foundation for the National Institutes of Health Biomarker Consortium Sarcopenia Project (FNIH) sex-specific cut-offs for weakness.

| **FNIH handgrip category** | **Cases/People at risk** | **Model 1**  **OR (95% CI)** | **Model 2**  **OR (95% CI)** |
| --- | --- | --- | --- |
| **Non-obese participants** |  |  |  |
| Normal | 173/2530 | 1.00 (reference) | 1.00 (reference) |
| Weak* | 34/239 | 1.82 (1.21, 2.74) | 1.56 (1.03, 2.37) |
| **Obese participants** |  |  |  |
| Normal | 102/984 | 1.58 (1.22, 2.05) | 1.38 (1.05, 2.80) |
| Weak* | 16/95 | 2.31 (1.31, 4.07) | 1.78 (0.99, 3.71) |

*Weakness defined as grip strength <26 kg (men); <16kg (women)

Model 1: comprises age and sex.

Model 2: comprises age, sex, physical activity, smoking, alcohol, wealth, time varying accumulative cardiovascular disease (angina, heart disease, heart failure, heart murmur, arrhythmia, stroke), diabetes, cancer, and arthritis.
